# Benchmarking desktop and mobile handwriting across COTS devices: The e-BioSign biometric database

**DOI:** 10.1371/journal.pone.0176792

**Published:** 2017-05-05

**Authors:** Ruben Tolosana, Ruben Vera-Rodriguez, Julian Fierrez, Aythami Morales, Javier Ortega-Garcia

**Affiliations:** Biometrics and Data Pattern Analytics (BiDA) Lab - ATVS, Universidad Autonoma de Madrid, Madrid, Spain; University of Texas at San Antonio, UNITED STATES

## Abstract

This paper describes the design, acquisition process and baseline evaluation of the new e-BioSign database, which includes dynamic signature and handwriting information. Data is acquired from 5 different COTS devices: three Wacom devices (STU-500, STU-530 and DTU-1031) specifically designed to capture dynamic signatures and handwriting, and two general purpose tablets (Samsung Galaxy Note 10.1 and Samsung ATIV 7). For the two Samsung tablets, data is collected using both pen stylus and also the finger in order to study the performance of signature verification in a mobile scenario. Data was collected in two sessions for 65 subjects, and includes dynamic information of the signature, the full name and alpha numeric sequences. Skilled forgeries were also performed for signatures and full names. We also report a benchmark evaluation based on e-BioSign for person verification under three different real scenarios: 1) intra-device, 2) inter-device, and 3) mixed writing-tool. We have experimented the proposed benchmark using the main existing approaches for signature verification: feature- and time functions-based. As a result, new insights into the problem of signature biometrics in sensor-interoperable scenarios have been obtained, namely: the importance of specific methods for dealing with device interoperability, and the necessity of a deeper analysis on signatures acquired using the finger as the writing tool. This e-BioSign public database allows the research community to: 1) further analyse and develop signature verification systems in realistic scenarios, and 2) investigate towards a better understanding of the nature of the human handwriting when captured using electronic COTS devices in realistic conditions.

## Introduction

Applications based on biometric user authentication have experienced a high deployment in many relevant sectors such as security, e-government, healthcare, education, banking or insurance in the last years [1]. This growth has been possible thanks to two main factors: 1) the technological evolution and the improvement of sensors quality [2], which have cut the prices of general purpose devices (smartphones and tablets) and therefore, the high acceptance of the society towards the use of them; and 2) the evolution of biometric recognition technologies in general [3–5]. However, the development and improvement of these biometric systems with very low error rates can not be possible without biometric databases with large amounts of realistic information.

**Fig 1:**
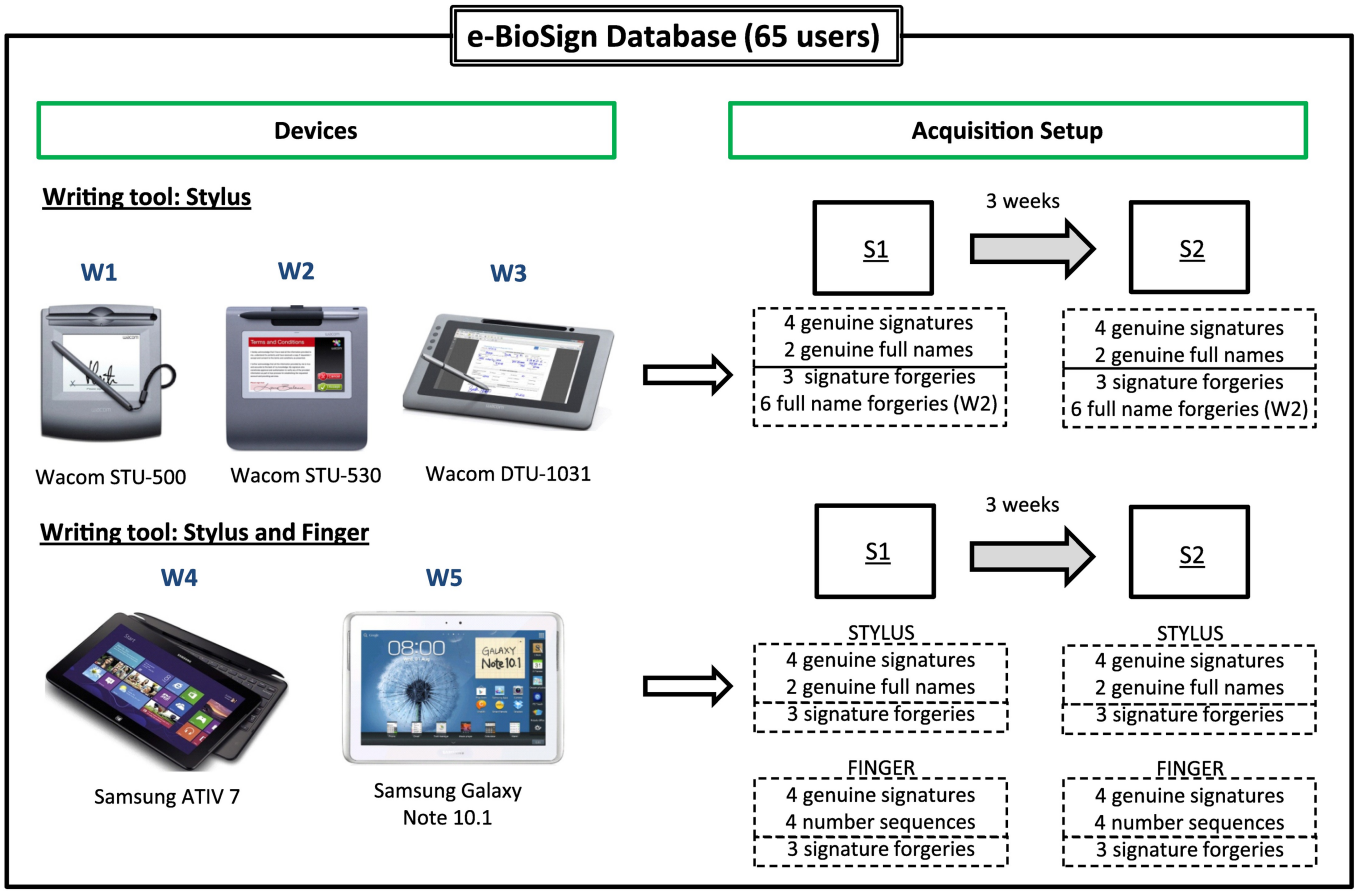
Description of the devices and the acquisition setup considered in the new e-BioSign database. A total of 65 users and 5 different COTS devices are considered (three Wacom and two Samsung general purpose devices). For the two Samsung devices, data is collected using both a pen stylus and also the finger.

This paper describes the design, collection and baseline evaluation of a new multi-scenario multi-device database called e-BioSign for dynamic signature and handwriting recognition (see Fig 1). This database is comprised of 65 users and data is collected in two sessions. It was designed to collect data from five COTS devices, three of them specifically developed for signature and handwriting applications (Wacom devices) and two general purpose tablets (Samsung tablets) that can collect data using both pen stylus and also the finger. We consider this last case as the “universal” case as everyone can have access to it. It is important to note that the devices considered in the e-BioSign database are some of the most common used devices in commercial, banking and e-health applications nowadays, so this database can encourage new lines of research for on-line signature recognition such as device interoperability and mixed writing-tool (pen stylus vs finger) recognition. Device interoperability scenarios have shown to be very challenging in terms of system performance in previous works [6]. However, it is important to highlight that previous studies were carried out considering devices not commercially available nowadays (e.g. PDAs). Therefore, a more up to date analysis of device interoperability scenarios considering current COTS devices should be carried out. The e-BioSign public database enables it. In addition, the mixed writing-tool scenario studied elsewhere [7] should be also updated considering more realistic setups as the one represented by e-BioSign.

Besides the research benefits in signature biometrics, the e-BioSign database also enables to investigate towards a better understanding of the nature of human handwriting [8], e.g., for e-health applications such as Parkinson disease [9] and brain strokes [10].

Additionally to the description of the e-BioSign database, this paper reports a benchmark evaluation for on-line signature verification. Two well-known feature extraction approaches (i.e. global and local systems [11]) followed in on-line signature verification are considered in order to further analyse the system performance over different scenarios. Three different scenarios representative of many current banking and commercial biometric applications are considered in the benchmark in order to cover a wide range of possible applications for on-line signature verification: 1) intra-device scenario (i.e. training and testing the systems using signatures acquired from the same device and writing tool), 2) inter-device scenario (i.e. training and testing the systems using signatures acquired from different devices but the same writing tool), and finally 3) mixed writing-tool scenario (i.e. training and testing the systems using signatures acquired from the same device but different writing tool).

Therefore, the main contributions of the present work are to describe the design and collection of the e-BioSign database and report a benchmark evaluation for on-line signature verification considering three different scenarios: 1) intra-device, 2) inter-device, and 3) mixed writing-tool. Also, the e-BioSign database is made freely available to the research community (https://atvs.ii.uam.es/atvs/eBioSign-DS1.html and http://dx.doi.org/10.6084/m9.figshare.4892600).

The remainder of the paper is organized as follows. In Section Related works, a review of the most relevant features of existing on-line signature databases is carried out pointing out the advantages of the e-BioSign public database presented in this work. Section e-BioSign database description describes the design and acquisition of the e-BioSign database. Section e-BioSign benchmark describes the experimental protocol and the results achieved. Finally, Section Conclusions draws the final conclusions and points out some lines for future work.

## Related works

Many efforts have been carried out in the signature biometrics community in order to capture large and reliable databases. In Table 1 the most relevant features of the main existing on-line signature databases are summarized. It is important to highlight the two largest databases (i.e. Biosecure [12] and BiosecurID [13]) with several hundreds of users, which are extensions of the largely used MCYT [14]. These three databases were collected by public institutions and have been extensively used by the signature research community for improving the state-of-the-art on many different scenarios [15]. However, the quality of the devices considered nowadays in the acquisition processes has improved significantly compared to those devices considered in [12–14]. Therefore, new databases with signatures acquired using COTS devices have appeared in the last years. One of the newer databases was presented in [16], where a total of 7 devices were used to acquire signatures. In that work, device interoperability was analysed only for random forgeries encouraging further research to improve the system performance. However, as far as we know, that database is not publicly available to the research community.

**Table 1:** Most relevant features of existing on-line signature databases.

	Year	Users	Sessions	#genuine samples/user/device	#forgeries/user/device	Device (writing tool)	Best performance (EER(%))
e-BioSign*	2016	65	2	8	6	Wacom STU-500 (stylus) Wacom STU-530 (stylus) Wacom DTU-1031 (stylus) Samsung Gal. Note (stylus/finger) Samsung ATIV7 (stylus/finger)	Stylus. Skilled: 7.9 Stylus. Random: 0.0 Finger. Skilled: 17.9 Finger. Random: 0.3
ATVS-SLT DB* [17, 18]	2015	29	6	46	10	Wacom Intuos 3 (stylus)	Stylus. Skilled: 1.4 [18] Stylus. Random: 0.0 [18]
ATVS-DooDB* [7]	2013	100	2	30	20	HTC Touch HD (finger)	Finger. Skilled: 21.0 [22] Finger. Random: 7.8 [22]
R.Blanco-Gonzalo *et al.* [16]	2013	43	3	60	-	Wacom Intuos 4 (stylus) Wacom STU-500 (stylus) Asus Eee PC Touch (stylus) Samsung Gal. Note (stylus/finger) BlackBerry Playbook (finger) Apple Ipad2 (finger) Samsung Gal. Tab (finger)	Stylus. Random: 0.58 [16] Finger. Random: 0.19 [16]
SUSIG* [23]	2009	100	2	20 (visual subcorpus) 8–10 (blind subcorpus)	10	Wacom Graphire2 (stylus) ePad-ínk (stylus)	Stylus. Skilled: 0.77 [24] Stylus. Random: 1.23 [24]
Biosecure* [12]	2008	667 (DS2) 713 (DS3)	2	30	20	Wacom Intuos3 (stylus) PDA HP iPAQ (stylus)	Stylus. Skilled: 6.2 [6] Stylus. Random: 2.0 [6]
BiosecurID* [13]	2007	400	4	16	12	Wacom Intuos3 (stylus)	Stylus. Skilled: 4.77 [25] Stylus. Random: 0.50 [25]
MBioID [26]	2007	120 (approx.)	2	20	-	Wacom Intuos2 (stylus)	-
R. Guest [19]	2006	274	variable	10–74	-	Graphic tablet (stylus)	-
MyIDEA* [27]	2005	104 (approx.)	3	18	18	Wacom Intuos2 (stylus)	Stylus. Skilled: 13.7 [28] Stylus. Random: 4.0 [28]
SVC2004* [29]	2004	100	2	20	20	Wacom Intuos (stylus) PDA (stylus)	Stylus. Skilled: 0.83 [24] Stylus. Random: 0.12 [24]
MCYT-100* [14]	2003	100	1	25	25	Wacom Intuos (stylus)	Stylus. Skilled: 2.85 [24] Stylus. Random: 1.04 [24]
BIOMET* [30]	2003	130 106 91	1	15	17	Wacom Intuos2 (stylus)	-

* publicly available databases.

Another related database that allows to study the aging problem in on-line signature is the ATVS On-Line Signature Long-Term database [17, 18]. In that database, signatures were acquired in six different sessions during a 15-month time interval. An assessment of the age dependency for on-line signature verification was performed in [19] considering a database with a total of 274 users. In [7], also both stylus and finger were considered as writing tools in the experimental work. For the finger case, users were asked to perform a simplified version of their signature (a.k.a. pseudo-signatures) based on their initials or part of their signature flourish. Results obtained in that preliminary work showed its feasibility and the necessity of further research toward practical application of such mixed input. In the present work, we make public to the research community a large signature database acquired from 5 different COTS devices in total, considering both pen stylus and also the finger. The complete design and acquisition of the e-BioSign database is described in Section e-BioSign database description. Additionally, new real scenarios such as the mixed-writing tool scenario not considered in the preliminary description of the e-BioSign database [20] are included in the experimental work of the present work (Section e-BioSign benchmark). For the near future, our idea is to extend e-BioSign by acquiring more signatures from the same users and writing tools in order to enable longer term studies on template aging for signature biometrics [17] and handwriting variability [21].

## e-BioSign database description

e-BioSign database is comprised of five handwriting capturing devices. Three of them are specifically designed for capturing handwritten data (Wacom devices), while the other two are general purpose tablets not designed for that specific task (Samsung tablets). Fig 1 shows an image of the setup used to acquire the database, with all five considered devices.

It is worth noting that all five devices were used with their own pen stylus. Additionally, the two Samsung devices were used with the finger as the writing tool, allowing us to analyse the effect of the writing tool on the system performance. The same capturing protocol was used for all five devices: they were placed on a desktop and subjects were told to feel comfortable when writing on them, so a small rotation of the devices was allowed.

The software for capturing handwriting and signatures was developed in the same way for all devices in order to minimize the variability of the user during the acquisition process. A rectangular area with an horizontal line in the bottom part were represented on the device screen, including two buttons “OK” and “Cancel” to press after writing if the sample was good or bad respectively. If the sample was not good, then it was repeated. The nomenclature and a brief description of each device considered in e-BioSign are given next:

1. ***W1: Wacom STU-500***. 5-inch TFT-LCD B/W display, with VGA resolution of 640 × 480 pixels. It has a sampling rate of 200 Hz, and 512 pressure levels. This device gives a very natural feel of writing.
2. ***W2: Wacom STU-530***. Newer version of W1 device. 5-inch TFT-LCD color display, with VGA resolution of 640 × 480 pixels. It has a sampling rate of 200 Hz, and 1024 pressure levels. This device allows safe transactions as it has AES 256 bit / RSA 2048 embedded data encryption.
3. ***W3: Wacom DTU-1031***. This device has a larger 10.1-inch color LCD display with a resolution of 1280 × 800 pixels. It has a sampling rate of 200 Hz, and 512 pressure levels. It also provides the same data encryption as W2. It allows to visualize documents on the display before signing them.
4. ***W4: Samsung ATIV 7***. This is a device with Windows 8. It has a 11.6-inch LED display with a resolution of 1920 × 1080 pixels. It has 1024 pressure levels, and contrary to the Wacom devices, the sampling rate is not uniform in this case. This tablet allows to use its own stylus or also the finger, but no pressure information is recorded in this last case.
5. ***W5: Samsung Galaxy Note 10.1***. This is an Android device. It has a 10.1-inch LCD display with a resolution of 1280 × 800 pixels. It has 1024 pressure levels and not uniform sampling rate. This device also allows to use its own stylus or the finger.

Table 2 shows the number of samples acquired for each user per session. As mentioned previously, the database was collected in two sessions with a time gap of at least three weeks between them. In each session there were three capturing blocks namely *Genuine 1*, *Genuine 2* and *Forgeries*. In *Genuine 1* block, two signatures plus the full name are performed for each device using their own pen stylus. Then, two signatures and a number sequence comprised of numbers from 0 to 9 plus a random letter are performed for the two Samsung devices with the finger. Next, *Genuine 2* block is recorded, which is comprised of the same information as *Genuine 1* block, but in this case the full name is written in capital letters. Finally, the last block *Forgeries* is performed, where each user carries out a forgery of the signatures of the three previous users in the database for each of the 5 devices using the stylus, and also with the finger for the two Samsung devices. Regarding forgeries of the full name, they are only performed for the Wacom STU-530 both for lower and upper case writing. In order to perform high quality forgeries, users are allowed to visualize a recording of the dynamic realization of the signature to forge.

**Table 2:** Handwritten samples captured in e-BioSign database per user and device in each of the two sessions.

	Block	Stylus	Finger
**Signature**	Genuine 1	2 (W1–W5)	2 (W4, W5)
	Genuine 2	2 (W1–W5)	2 (W4, W5)
	Forgeries	3 (W1–W5)	3 (W4, W5)
**Full name**	Genuine 1	1 (W1–W5)	-
	Forgeries	3 (only W2)	-
**Full name capital letters**	Genuine 2	1 (W1–W5)	-
	Forgeries	3 (only W2)	-
**Number sequence**	Genuine 1	-	2 (W4, W5)
	Genuine 2	-	2 (W4, W5)

In the second session, the procedure is identical, except one difference in the *Forgeries* block. In this case, users forge the same users as in session one, but this time a paper with the image of the signatures and names to forge is placed over the screen devices so the users can overwrite to perform the forgeries. Nevertheless, they are not allowed to see the recordings of the signatures in this case.

In total there are 6,370 signatures, of which 3,640 are genuine samples and 2,730 are forgeries. From the total, 4,550 were performed with the stylus and 1,820 with the finger. There are a total of 2,080 handwritten names, of which 1,300 are genuine samples and 780 are forgeries (only for Wacom STU-530). Also, half of the samples are done with natural writing and the other half in capital letters. Finally, there are 1,040 genuine alphanumeric sequences carried out for the two Samsung devices using the finger.

**Fig 2:**
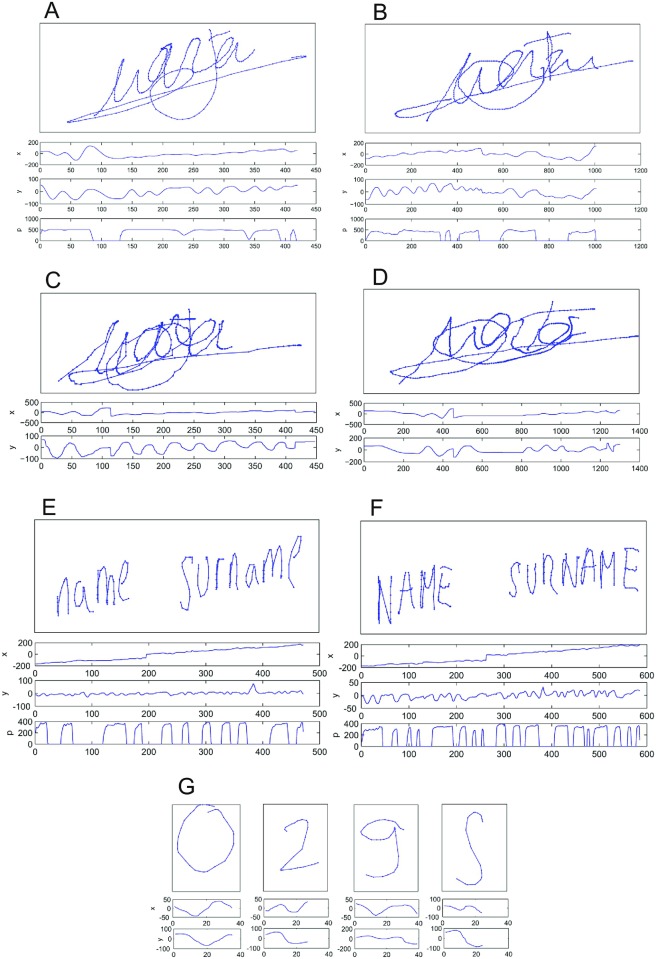
Example of the data collected in e-BioSign database for Samsung Galaxy Note 10.1. (A) Genuine signature pen stylus. (B) Forgery signature pen stylus. (C) Genuine signature finger. (D) Forgery signature finger. (E) Name lower-case. (F) Name upper-case. (G) Number sequence.

The whole capturing process was supervised by an operator who explained all the steps that donors had to follow. Therefore, this is a multi-session and multi-device database with samples captured using both pen stylus and finger as the writing tools for signature and handwritten data. Fig 2 shows examples of the data collected in e-BioSign for the Samsung Galaxy Note 10.1 (W5), as this device contains all types of information collected, i.e., signatures (genuine and forgeries) using the pen stylus and the finger, full name in lower and upper cases (only genuine as the forgeries were only performed for the Wacom STU-530) and number sequences made with the finger. Fig 2E and 2F are just examples in order not to reveal the name of any user of the database. The rest of the samples are contained in the database. It is worth noting that data collected using the finger for Samsung ATIV 7 and Galaxy Note 10.1 do not contain pressure information as this was not provided by these devices, and there is also no information of the trajectory (*X* and *Y* coordinates) during pen-ups. For the case of signatures acquired using the pen stylus, pressure information and pen-up trajectories are available for all devices and have been used in the evaluation reported in this paper with the exception of the mixed writing-tool scenario (Section **Experiment 3: mixed writing-tool analysis**).

**Fig 3:**
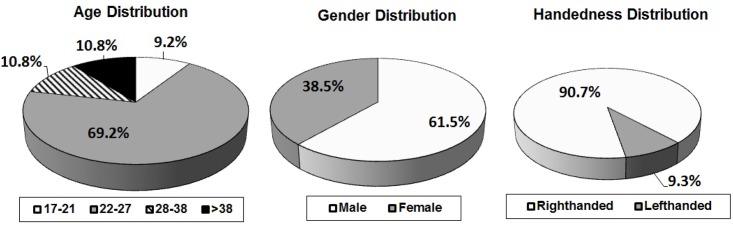
Population statistics of e-BioSign database.

Fig 3 shows the statistics of the population of e-BioSign database. Regarding the age distribution, the majority of the subjects (69.2%) are between 22 and 27 years old, as the database was collected in a university environment. Fig 3 also shows the handedness and the gender distributions. The gender was designed to be as balanced as possible, having 61.5% of males and 38.5% of females. Regarding the handedness distribution, 90.7% of the population is righthanded.

## e-BioSign benchmark

This section reports the benchmark evaluation carried out for on-line signature verification on the e-BioSign database. Section Baseline systems describes the two reference verification systems considered. Then, Section Experimental protocol gives the experimental details of the three scenarios considered for the benchmark evaluation. Finally in Section Experimental results the results obtained are analysed.

### Baseline systems

Two well-known approaches for feature extraction in on-line signature verification are evaluated [11]: feature-based (a.k.a. global system) and time functions-based (a.k.a. local system). For each signature acquired using the stylus, signals related to *X* and *Y* coordinates and pressure are used to extract a set of 117 features and 23 time functions for global and local systems respectively whereas for signatures acquired using the finger, only features and time functions related to *X* and *Y* coordinates are considered as pressure information is not available. Information related to pen angular orientation (azimuth and altitude angles) has been always discarded in order to consider the same set of features and time functions that we would be able to use in general purpose devices such as tablets and smartphones. The feature/time-function selection SFFS algorithm [31] has been performed over a development dataset in order to obtain optimal features and time-functions subsets for each scenario, improving the performance of the system in terms of EER (%).

The considered global and local systems are based on previous studies [6, 11, 31, 32]. For the global system, the Mahalanobis distance algorithm is used to compute the similarity between the input signature and the claimed user model whereas for the local system, the DTW algorithm [11] is used to compute the similarity between the time functions from the input and training signatures.

### Experimental protocol

The experimental protocol has been designed to cover a wide range of possible banking and commercial applications for on-line signature verification. Three main experiments are carried out in this work: in **Experiment 1: intra-device analysis**, the system performance of each device is analysed considering the scenario of having signatures from the same device and writing tool. This analysis is performed for both global and local systems. In **Experiment 2: inter-device analysis**, the scenario of considering signatures from different devices but same writing tool for training and testing the system is studied. Finally, in **Experiment 3: mixed writing-tool analysis**, we evaluate the performance of the local systems in the challenging scenario of considering signatures acquired by the same device but different writing tools (i.e. stylus or finger) for training and testing the systems.

The e-BioSign database is divided into two different datasets, one for development and training the systems and the other one for evaluation. The first 30 users of the e-BioSign database are used in the development and training phase for both global and local systems while the remaining 35 users are considered in the evaluation phase.

Regarding the development and training phase, different experimental protocols have been proposed for each of the three considered scenarios. All details about the training procedures are given for each experiment in the next section.

Regarding the evaluation phase, the same experimental protocol is considered for all experiments. The 4 genuine signatures of the first session are used as reference signatures, whereas the remaining 4 genuine signatures of the second session are left for testing. Skilled forgery scores are obtained by comparing the reference signatures against the 6 available skilled forgeries per user whereas random (zero-effort) forgery scores are obtained by comparing the reference signatures with one genuine signature of each one the remaining users. For the global system, scores are obtained by comparing test signatures against the user model obtained with the 4 reference signatures whereas for the local system, the average score of the four one-to-one comparisons is performed.

### Experimental results

#### Experiment 1: Intra-device analysis

In this experiment, an evaluation of the e-BioSign database is carried out for both global and local systems considering an intra-device scenario with signatures acquired using the same writing tool. Two different approaches are considered in this experiment. First, Baseline systems whose features/time-functions are fixed from previous works (see Tables 3 and 4). Second, the global and local systems are adjusted in order to improve the system performance for each device by using the set of features/time-functions selected by the SFFS algorithm on the 30 users of the development dataset (starting from the whole set of 117 global features and 23 time functions). In order to do that, the 4 genuine signatures of the first session are used as training signatures, whereas the 4 genuine signatures of the second session are left for testing in order to consider inter-session variability. The feature/time-function selection SFFS algorithm has been individually applied to each device and writing tool. This second approach will be considered as Proposed.

**Table 3:** Experiment 1: Features considered in the global baseline system. Feature # taken from [32, 33].

#	Feature description
1	Signature total duration Ts
2	N(pen-ups)
36	(*x*_*max*_ − *x*_*min*_) / *x*_*acquisition*__*range*_
67	(*y*_*max*_ − *y*_*min*_) / *y*_*acquisition*__*range*_
101	Average pressure $\bar{p}$

**Table 4:** Experiment 1: Time functions considered in the local baseline system. Time-function # taken from [18].

#	Time-function description
1	x-coordinate: *x*_*n*_
2	y-coordinate: *y*_*n*_
8-9	First-order derivate of features 1-2: ${\dot{x}}_{n},{\dot{y}}_{n}$
15-16	Second-order derivate of features 1-2: ${\ddot{x}}_{n},{\ddot{y}}_{n}$
19	First order difference of angle of consecutive samples: ${\dot{\alpha }}_{n}$

Tables 5 and 6 show results for both Baseline and Proposed approaches considering the 35 users of the evaluation dataset. The global system is only considered for the case of using the stylus as the writing tool (Table 6) as information related to pen ups and pressure is not available in the W5 device when signatures are acquired using the finger.

**Table 5:** Experiment 1 (Intra-device scenario): System performance results (EER in %) for the local systems when signatures are acquired using stylus and finger. B = Baseline and P = Proposed.

	STYLUS										FINGER			
	W1		W2		W3		W4		W5		W4		W5	
	B	P	B	P	B	P	B	P	B	P	B	P	B	P
Skilled	10.0	8.3	10.0	10.0	15.7	13.6	10.0	7.9	12.9	10.7	24.0	22.1	27.0	26.4
Random	1.4	0.0	1.1	0.7	4.3	2.9	0.8	0.7	2.1	1.0	1.4	0.3	2.3	1.0

**Table 6:** Experiment 1 (Intra-device scenario): System performance results (EER in %) for the global systems when signatures are acquired using stylus. B = Baseline and P = Proposed.

	STYLUS									
	W1		W2		W3		W4		W5	
	B	P	B	P	B	P	B	P	B	P
Skilled	13.6	13.5	12.9	16.4	22.6	19.3	14.3	17.9	19.3	10.0
Random	12.1	10.7	12.1	13.6	20.8	17.9	11.4	12.1	17.9	6.4

Analysing Table 5 for the case of using the stylus as the writing tool, the baseline systems achieve an average EER of 11.7% and 1.9% for skilled and random forgery cases respectively whereas for the proposed systems the average EER improves to 10.1% and 1.1% for skilled and random forgeries respectively. These results show the benefits of using the time-function selection SFFS algorithm over a development dataset. In addition, two important observations can be highlighted from the results. First, very similar system performance has been achieved for general purpose devices (i.e. W4 and W5) compared to devices specifically designed to capture dynamic signatures (i.e. W1, W2 and W3) for both skilled and random forgery cases when the stylus is used as the writing tool. This shows the feasibility of general purpose devices in real banking and commercial applications. Second, it is worth noting a lower performance of the W3 device compared to the other devices even being a high quality Wacom device. We attribute this fact to the the user interface as during the acquisition process, which included a cross shape marker was included on the display in contrast to the other devices, and this could have been uncomfortable for the users.

Analysing Table 5 for the case of using the finger as the writing tool, the baseline systems achieve an average EER of 25.5% and 1.9% for skilled and random forgery cases respectively whereas for the proposed systems the average EER improves to 24.3% and 0.7% for skilled and random forgeries respectively, showing again the benefits of using the time-function selection SFFS algorithm. An important effect that can be observed from Table 5 is how the system performance changes regarding the writing tool (i.e. stylus and finger) considered during the acquisition process. Analysing the results of Table 5 for W4 and W5 proposed systems, an average EER of 9.3% and 0.9% is achieved when signatures are acquired using the stylus whereas for the finger case, the average EER is 24.3% and 0.7%. It is important to note the high EER obtained for the skilled forgery cases when the finger is considered as the writing tool (almost three times worse than the stylus case). The reason for this effect can be due to the very challenging scenario considered for the finger case as forgers had access to the dynamic realization of the signatures to forge. A recommendation for the usage of signature recognition on mobile devices would be for the users to protect themselves from other people that could be watching while signing as this is more feasible to do in a mobile scenario compared to an office scenario. This way skilled forgers might have access to the global shape of a signature but not to the dynamic information. Therefore, the use of the finger as the writing tool for real commercial and banking applications seems to be feasible, signatures acquired using the finger seem to be a real possibility for many commercial and banking applications specially for random forgery cases where even better results are achieved compared to the stylus case.

Analysing Table 6, the average EER for the baseline systems is 16.5% and 14.8% for skilled and random forgery cases respectively whereas for the proposed systems the average EER is 15.4% and 12.1% for skilled and random forgery cases respectively. Therefore, the use of the global system does not achieve as good results as the local system. In particular, the results obtained for random forgery cases are much worse compared to the local system results. For this reason the global system has been discarded in the next experiments.

Finally, it is important to compare the best system performance for e-BioSign database to the best system performances from other existing on-line signature databases (see Table 1). However, it is worth noting that it is not possible to make a comparative analysis between all databases because different experimental protocols and signature verification systems are used in each work. For the case of using the stylus as the writing tool, the best results obtained in the present work are 7.9% and 0.0% EER for skilled and random forgeries cases respectively. The result obtained for skilled forgeries is a bit higher in terms of EER compared to the results obtained in the two largest databases (i.e. 6.20% and 4.77% EER for Biosecure and BiosecurID databases respectively). One of the possible reasons for this effect could be the high quality of the forgeries from e-BioSign database as forgers could even place on the screen device a paper with the image of the signatures to forge. For the random forgeries case, the result achieved here is 0.0% EER, which is the best compared to other databases. This could be due to usage of COTS devices for real applications. Analysing the case of using the finger as the writing tool, the best results obtained are 17.9% and 0.3% EER for skilled and random forgeries respectively. For the skilled forgeries case, the result obtained using the e-BioSign database has outperformed the preliminary results obtained in [22], in which users were asked to perform a simplified version of their signature (a.k.a. pseudo-signatures) based on their initials or part of their signature flourish. For the random forgeries case, the result obtained is very close to zero, similar to the result obtained in [16].

#### Experiment 2: Inter-device analysis

In this experiment the main goal is to evaluate the system performance considering an inter-device scenario (i.e. signatures from different devices but the same writing tool are used for training and testing the system). The approach followed in [6] is applied in this experiment in order to compensate for device interoperability. This approach is based on two main stages. The first one is a preprocessing stage where data acquired from different devices are processed in order to normalize the signals to similar ranges. The second stage is based on the use of time-function selection via SFFS in order to select the most robust time functions for device interoperability scenarios. Two different systems are developed for all five devices (one for signatures acquired using the stylus and another one for the finger) using the 30 users of the development dataset. In order to develop the systems, for the stylus a total of 5 genuine signatures (1 signature per device) of the first session are considered as training signatures whereas a total of 20 genuine signatures (4 signatures per device) of the second session are left for testing. For the finger case a total of 4 genuine signatures (2 signatures per device) of the first session are considered as training signatures whereas the 8 genuine signatures (4 signatures per device) of the second session are left for testing. Then the systems developed were tested on the 35 users of the evaluation dataset. Results achieved are depicted in Tables 7 and 8.

**Table 7:** Experiment 2 (Inter-device scenario): System performance results (EER in %) for the local proposed system when signatures are acquired using a pen stylus. Skilled and random forgeries results are shown on top and bottom of each cell respectively.

		Test				
		W1	W2	W3	W4	W5
Train	W1	10.7 0.7	7.9 0.8	15.7 5.0	10.7 0.7	10.7 2.1
	W2	11.4 1.1	10.0 0.7	16.4 5.7	14.3 0.7	11.4 1.6
	W3	9.3 0.3	8.6 0.7	13.6 2.1	11.2 0.0	11.4 1.4
	W4	10.0 0.7	9.3 0.9	17.1 5.0	10.7 0.7	11.4 1.4
	W5	12.7 1.4	10.0 1.1	16.9 5.0	12.1 0.7	11.2 1.4

**Table 8:** Experiment 2 (Inter-device scenario): System performance results (EER in %) for the local proposed system when signatures are acquired using the finger. Skilled and random forgeries results are shown on top and bottom of each cell respectively.

		Test	
		W4	W5
Train	W4	19.3 0.7	23.5 0.2
	W5	24.2 0.7	22.9 0.3

Table 7 shows all possible device combinations for training and testing the systems when the stylus is used as the writing tool. The diagonal of Table 7 (highlighted in darker colour) contains all results without device interoperability. The proposed system developed for this scenario achieves an average EER for device interoperability cases of 11.9% and 1.8% for skilled and random forgery cases respectively whereas for no device interoperability cases the average EER is 11.2% and 1.1% for skilled and random forgery cases respectively. Therefore, very similar results have been achieved in general with and without device interoperability when the pen stylus is considered as the writing tool. These results show the importance of applying device interoperability compensation techniques. It is also important to note the system performance obtained when W3 device is used for testing the system. For these cases, the average EER increases up to 15.9% and 4.6% for skilled and random forgery cases respectively. This degraded performance could have been produced due to the same reasons explained in the previous experiment.

Table 8 also shows all possible device combinations for training and testing the system when the finger is considered as the writing tool. The proposed system developed for this scenario achieves an average EER for the device interoperability cases of 23.9% and 0.5% for skilled and random forgeries respectively whereas for no device interoperability the average EER is 21.1% and 0.5% for skilled and random forgeries respectively. Therefore, the same observations previously extracted for the stylus case can be also applied here when writing with the finger in a general mobile device.

Finally, some important conclusions can be extracted comparing the results obtained in this work to the results obtained in previous work [6]. The high technological evolution and the improvement of sensor quality together with our proposed approach for dealing with device interoperability lead to very competitive signature verification systems based on finger input over COTS smartphones.

#### Experiment 3: Mixed writing-tool analysis

In this experiment the main goal is to explore a new scenario where on-line signature verification systems are trained and tested using signatures from the same device but acquired from different writing tools (i.e. stylus and finger). This scenario can be very useful for many banking and commercial applications where the user first register in the system using a pen stylus and then in posterior usages they could make use of their personal smartphone or tablet devices using the finger as the writing tool. Two different local systems are considered in this experiment, one for W4 device and another one for W5 device. The time-function selection SFFS algorithm is applied using the 30 users of the development dataset in order to select the most robust functions for mixed writing-tool scenarios. SFFS has been individually applied to W4 and W5 devices considering a total of 4 genuine signatures (2 signatures per writing tool) as training signatures and 8 genuine signatures (4 signatures per writing tool) of the second session as testing signatures. Table 9 shows the results obtained for each device in this mixed writing-tool scenario considering the 35 users of the evaluation dataset.

**Table 9:** Experiment 3 (Mixed writing-tool scenario): System performance results (EER in %) when the local proposed systems are trained and tested using signatures from the same device but different writing tools (i.e. stylus and finger). Skilled and random forgeries results are shown on top and bottom of each cell respectively.

		Test			
		W4		W5	
		Stylus	Finger	Stylus	Finger
Train	Stylus	12.9 0.7	22.9 0.7	12.9 0.1	17.9 0.2
	Finger	27.9 0.7	20.0 0.7	18.6 0.7	17.9 0.5

Analysing the skilled forgery cases, results show in general a system performance degradation when signatures acquired using the finger are considered for training or testing the systems. For this case, an average 19.0% EER is achieved when the finger is considered for both training and testing the system whereas for the mixed writing-tool scenario the average EER is 21.8%. Therefore, although the system performance is slightly worse for the mixed writing-tool scenarios, the main problem resides in the signatures acquired with the finger, case that will be deeply studied in our future work. In addition, it is important to note that for both devices the worst mixed writing-tool case seems to be when the local system is trained using signatures acquired through the finger and testing with the stylus but this would not be a common application scenario.

Analysing the random forgery cases, similar results can be observed for both devices when mixed and no mixed writing-tool scenarios are considered. Therefore, the deployment of signature verification in real applications on the new and challenging finger and mixed writing-tool scenarios seems to be feasible when random forgeries are considered.

As a conclusion of the results depicted in Table 9, an exhaustive analysis of this very challenging scenario needs to be carried out in future works for skilled forgery cases.

## Conclusions

This paper has described the design, acquisition process and baseline evaluation of the new e-BioSign on-line signature and handwriting database. This database is comprised of 5 COTS devices in total. Three of them are Wacom devices (STU-500, STU-530 and DTU-1031) specifically designed to capture dynamic signatures and handwriting, and the other two are Samsung general purpose tablets (Samsung Galaxy Note 10.1 and Samsung ATIV 7). For the two Samsung tablets, data is collected using both pen stylus and finger in order to study the performance of signature verification systems and handwriting in general in a mobile scenario. Data was collected in two sessions for 65 subjects, including dynamic information of the signature, the full name and number sequences. Skilled forgeries were also performed for signatures and full names.

It is important to note that, as far as we know, this is the first publicly available on-line handwriting database that allows the research community to make an analysis of very important and challenging scenarios for signature biometrics and handwriting such as inter-device and mixed writing-tool considering COTS devices. The e-BioSign database also enables future research towards a better understanding of the human handwriting when captured using electronics COTS devices in realistic conditions.

Together with the e-BioSign description, a benchmark evaluation has been defined to evaluate signature biometric technologies under different realistic scenarios currently present in diverse banking and commercial applications: 1) intra-device, 2) inter-device, and 3) mixed writing-tool scenarios. The results obtained in the first scenario have shown a significant difference of performance in skilled forgery cases for signatures acquired with a pen stylus (average of 10.1% EER) compared to signatures acquired using the finger (average of 24.3% EER). Nevertheless, when considering random forgeries, the results achieved with the finger as writing tool are even better than using the stylus (below 1.0% EER). Regarding the inter-device scenario, the evaluated algorithms including data preprocessing and feature selection have demonstrated to be robust against device interoperability. Finally, the results obtained for skilled forgery cases considering the mixed writing-tool scenario are similar to those achieved for the case of using the finger. For future work, an exhaustive analysis of the system performance for skilled forgery cases will be carried out for both pen stylus and finger writing tools. Additionally, e-BioSign database will be extended with more users and more sessions in time in order to make it feasible to conduct research on template aging for signature and handwriting biometrics.
